# Do Ames dwarf and calorie-restricted mice share common effects on age-related pathology?

**DOI:** 10.3402/pba.v3i0.20833

**Published:** 2013-06-20

**Authors:** Yuji Ikeno, Gene B. Hubbard, Shuko Lee, Sara M. Dube, Lisa C. Flores, Madeline G. Roman, Andrzej Bartke

**Affiliations:** 1 The Barshop Institute for Longevity and Aging Studies, San Antonio, TX, USA; 2 Department of Pathology, The University of Texas Health Science Center at San Antonio, San Antonio, TX, USA; 3 Research Service, Audie L. Murphy VA Hospital (STVHCS), San Antonio, TX, USA; 4 Department of Internal Medicine, Southern Illinois University School of Medicine, Springfield, IL, USA

**Keywords:** age-related pathology, Ames dwarf mice, calorie restriction, neoplastic disease, aging

## Abstract

Since 1996, aging studies using several strains of long-lived mutant mice have been conducted. Among these studies, Ames dwarf mice have been extensively examined to seek clues regarding the role of the growth hormone/insulin-like growth factor-1 axis in the aging process. Interestingly, these projects demonstrate that Ames dwarf mice have physiological characteristics that are similar to those seen with calorie restriction, which has been the most effective experimental manipulation capable of extending lifespan in various species. However, this introduces the question of whether Ames dwarf and calorie-restricted (CR) mice have an extended lifespan through common or independent pathways. To answer this question, we compared the disease profiles of Ames dwarf mice to their normal siblings fed either *ad libitum* (AL) or a CR diet. Our findings show that the changes in age-related diseases between AL-fed Ames dwarf mice and CR wild-type siblings were similar but not identical. Moreover, the effects of CR on age-related pathology showed similarities and differences between Ames dwarf mice and their normal siblings, indicating that calorie restriction and Ames dwarf mice exhibit their anti-aging effects through both independent and common mechanisms.

Ames dwarf (*Prop1 df*) mice have drawn much attention in aging research because of their marked life extension (1, 2). Although studies demonstrate that some of the physiological characteristics of Ames dwarf mice are similar to those of calorie-restricted (CR) mice (2, 3), it is still unclear whether these mice share common underlying mechanisms for their anti-aging traits. We previously reported that Ames dwarf mice have a delayed occurrence of fatal neoplastic disease compared to their wild-type siblings (4). To further investigate whether Ames dwarf and CR mice have extended lifespans by similar or independent mechanisms, we compared the disease profiles of Ames dwarf mice and their normal siblings fed either *ad libitum* (AL) or a CR diet. Here we report our findings that the changes in age-related diseases between AL-fed Ames dwarf mice and CR normal siblings were similar but not identical. Moreover, the effects of CR on age-related pathology showed similarities and differences in Ames dwarf mice and their normal siblings, indicating that both independent and common mechanisms are involved in their anti-aging effects.

The Ames dwarf mice used in this study were derived from mating male dwarfs (*df*/*df*) with carrier females (*df/+*). To examine the age-related disease profiles, 2-month-old Ames dwarf (*df/df*) mice and their normal siblings were divided into two groups, AL and CR. The basic operations of the animal colony were those previously described (5, 6). The mice in the CR group were fed every day, with food intake reduced in successive weeks to 90%, 80%, and finally 70% of the daily consumption of genotype- and sex-matched AL animals. Because the food consumption of AL mice declines naturally with age, the amount of food given to CR mice was kept constant after 24 months of age. The pathology of 126 mice was examined: 60 Ames dwarf mice (41 AL and 19 CR) and 66 normal siblings (43 AL and 23 CR). After complete gross pathological examination, the tissues were excised, and the fixed tissues were processed conventionally and embedded in paraffin, sectioned at 5 µm, and stained with hematoxylin-eosin for histopathological analysis.

The survival curve of Ames dwarf mice was previously shown (6). The average, median, and maximum (10%) lifespans of the mice in this study are shown in Table 1. The AL Ames dwarf and the CR wild-type mice showed significantly longer lifespans (average, median, and maximum lifespans) compared to the AL wild-type mice (*p*≤0.01). There was no significant difference in the average, median, and maximum lifespans of AL Ames dwarf mice compared to CR wild-type mice. Moreover, the CR Ames dwarf mice showed a further extension of the average, median, and maximum lifespans compared to AL Ames dwarf mice and CR wild-type mice (*p*≤0.01).

**Table 1:** Summary of longevity

	Dwarf		WT	
		
Lifespan (days)	AL (*n*=41)	CR (*n*=19)	AL (*n*=43)	CR (*n*=23)
Average	999*	1,134.6**	714.2	869.4*
Median (50%)	995*	1,230**	732.5	917*
Maximum	1,299*	1,458**	889	1,184*

* *p*<0.01

** *p*<0.01.

The probable causes of death in the dwarf and wild-type groups are shown in Table 2. Approximately 90% of AL wild-type mice died from neoplastic diseases, the majority of which (approximately 42%) were pulmonary adenocarcinomas (Fig. 1A). The other major fatal neoplastic diseases that we observed were hepatocellular carcinoma (HCC), lymphoma, and hemangioma in the liver and spleen, (Fig. 1B, 1C and 1D, respectively). These fatal neoplastic diseases were usually associated with metastasis to other organs or other pathological lesions, e.g., pleural effusion, ascites, hemorrhage, or severe congestion and edema in the lung. The AL dwarf mice showed a slightly lower incidence of fatal neoplasms, while the incidence of fatal pulmonary adenocarcinoma was significantly lower in these mice compared to AL wild-type mice (*p*≤0.012). Although the incidence of fatal HCC was slightly higher in the AL dwarf mice, it was not significantly different from AL wild-type mice and occurred at an older age after a majority of the AL wild-type mice had died. The CR wild-type mice showed significantly lower incidences of fatal neoplasms compared to AL wild-type mice (*p*≤0.04), but the incidence of fatal pulmonary adenocarcinoma was not reduced in CR normal siblings, which is different than the data from AL dwarf mice. Approximately 20% of AL dwarf mice and 25% of CR wild-type mice died without obvious evidence of lethal pathological changes (unknown cause of death), which could indicate that tissue integrity was maintained in these mice during aging. The incidence of unknown cause of death was significantly higher in the CR wild-type than the AL wild-type group (*p*≤0.03). However, the incidence of unknown cause of death in the AL Ames dwarf mice was not statistically significant compared to the AL wild-type group (*p*≤0.06). The CR Ames dwarf mice showed a significantly lower incidence of fatal neoplasms compared to AL wild-type siblings (*p*≤0.02); however, we found that the incidence of fatal neoplastic lesions was not reduced compared to AL Ames dwarf mice or CR wild-type mice. Interestingly, CR Ames dwarf mice did not show significant differences in the incidence of fatal pulmonary adenocarcinoma compared to the AL wild-type mice, which differs from the AL Ames dwarf mice. Furthermore, the incidence of fatal HCC was slightly lower in CR Ames dwarf mice compared to AL Ames dwarf mice, and CR Ames dwarf mice had a higher incidence of deaths that were without obvious evidence of lethal pathological changes compared to AL wild-type mice (*p*≤0.0002), indicating that CR has additive anti-aging effects in Ames dwarf mice.

**Fig. 1:**
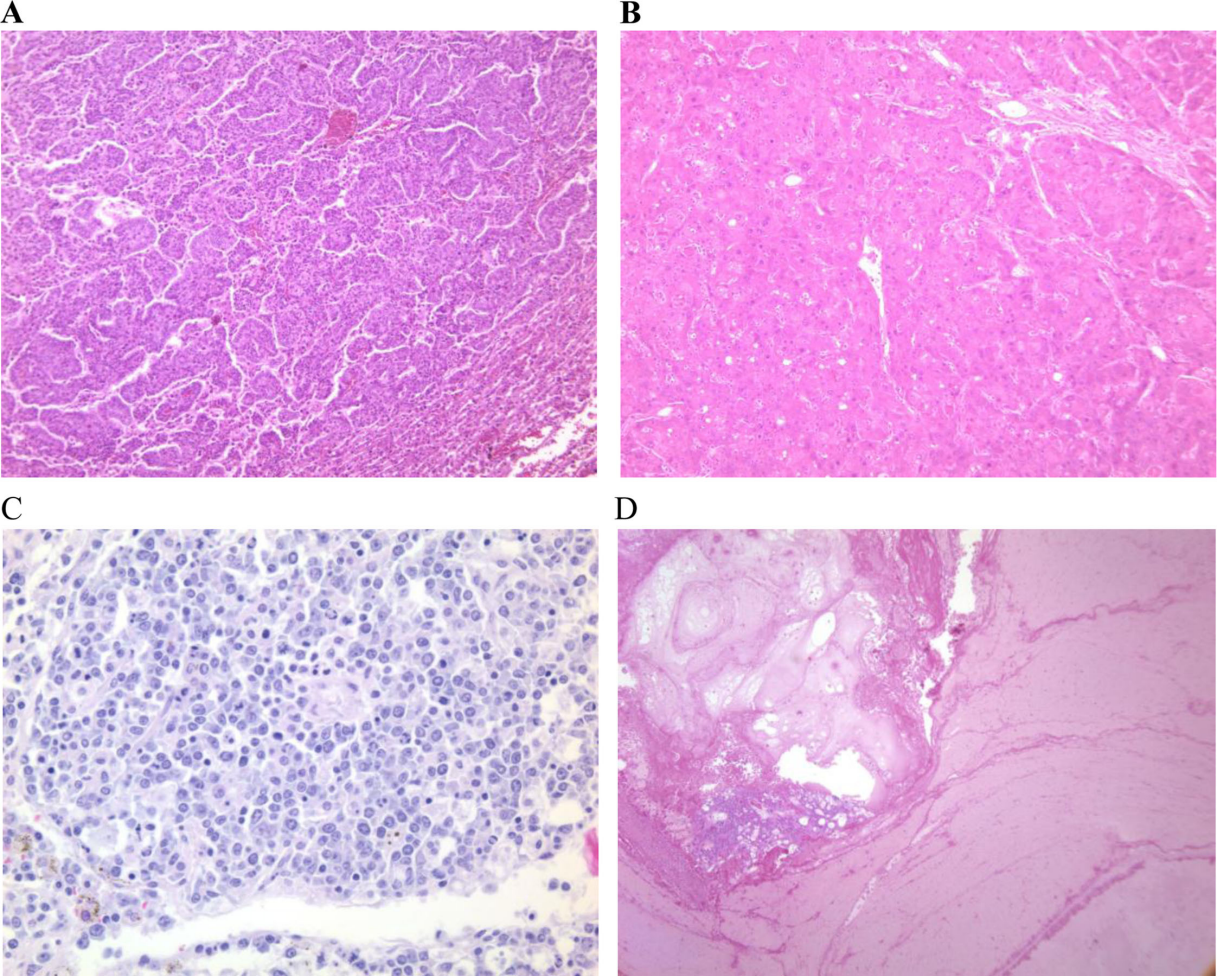
Histology of major pathology in Ames dwarf and wild-type mice. Major and potentially fatal pathological changes observed in Ames dwarf and wild-type mice are shown: (A) pulmonary adenocarcinoma, HE×100; (B) hepatocellular carcinoma, HE×100); (C) lymphoma, HE×400; and (D) hemangioma, HE×40.

**Table 2:** Cause of death (*df*/*df* mice)

	Dwarf		WT	
		
	AL (*n*=41)	CR (*n*=19)	AL (*n*=43)	CR (*n*=23)
Neoplasm	31 (75.6%)	10 (52.6%)*	39 (90.7%)	14 (60.9%)*
Adenocarcinoma	5 (12.2%)*	5 (26.3%)	18 (41.9%)	7 (30.4%)
HCC	13	2	5	0
Hemangioma	6	3	6	4
Lymphoma	4	0	8	3
Others	3	0	2	0
Non-neoplasm	10 (24.4%)	9 (47.4%)	4 (9.3%)	9 (39.1%)
Thrombus	1	0	0	0
Glomerulonephritis	0	0	3	1
Others	1	0	0	2
Unknown	8	9*	1	6*

* *p*<0.05.

When we compared differences in fatal neoplastic diseases between groups using Kaplan–Meier survival curves, we found that the patterns of the AL Ames dwarf and CR normal siblings showed a shift toward older ages compared to those of the AL normal siblings. Furthermore, the survival patterns of the CR Ames dwarf mice showed a shift toward an older age than patterns for mice in AL Ames dwarf and CR normal siblings. These data suggest that fatal neoplastic disease occurred at an older age for AL Ames dwarf mice and CR normal siblings than the AL normal siblings and that CR dwarf mice had a further delay in the occurrence of fatal neoplastic lesions.

The pathology data from this study indicate that long-lived Ames dwarf mice are not merely mimics of CR mice but that Ames dwarf and CR mice have both independent and shared underlying mechanisms for extended longevity. A study that compared gene expression patterns also showed a partial overlap between CR and Snell dwarf mice, suggesting that there are common and independent mechanisms in their anti-aging action as well (7). A possible common underlying mechanism shared by Ames dwarf and CR mice is a change in the growth hormone (GH)/insulin-like growth factor 1 (IGF-1) axis (2, 3, 6). The reduced levels of GH and the resulting suppression of peripheral IGF-1 levels are associated with significant life extension in Ames dwarf and CR mice and are also seen in animals that have a targeted disruption of the GH receptor (GHR) that results in GH resistance and severely suppressed IGF-1 levels (7). Endocrine changes in Ames dwarf and CR mice have also been found to lead to reductions in plasma insulin and glucose levels and in somatic growth and adult body size (8). The reduction of plasma IGF-1 and the reduced mitogenic actions of IGF-1 may be related to the delayed occurrence of fatal neoplastic disease observed in both AL Ames dwarf and CR wild-type mice due to the substantial evidence suggesting that GH and IGF-1 levels play important roles in tumor development through their potent mitogenic and anti-apoptotic effects (9). The pathological profiles of AL Ames dwarf and CR wild-type mice also showed some differences. CR wild-type mice had a reduced incidence of fatal neoplastic disease; however, AL Ames dwarf mice did not show a reduction in incidence of fatal neoplastic lesions, except adenocarcinoma in the lung. CR Ames dwarf mice showed a similar pathological profile to CR wild-type mice and had other additive effects on age-related pathology. These data agree with previous results that show that CR Ames dwarf mice have additive effects on the expression of a subset of genes (10) as well as a further extension of longevity. Further study is required to identify the underlying mechanisms that are independently involved in the anti-aging action of CR and Ames dwarf mice.

We therefore suggest that a common underlying mechanism in Ames dwarf and CR mice is the change in the endocrine system, especially in GH and IGF-1 levels, and that the subsequent pathophysiological changes play important roles in the delay of various age-related pathologies and in life extension. However, the possible independent mechanisms that extend lifespan and change age-related pathology in these mice remain unidentified.
